# A Network Meta-Analysis on Randomized Trials Focusing on the Preventive Effect of Statins on Contrast-Induced Nephropathy

**DOI:** 10.1155/2014/213239

**Published:** 2014-09-07

**Authors:** Mariangela Peruzzi, Leonardo De Luca, Henrik S. Thomsen, Enrico Romagnoli, Fabrizio D'Ascenzo, Massimo Mancone, Gennaro Sardella, Luigi Lucisano, Antonio Abbate, Giacomo Frati, Giuseppe Biondi-Zoccai

**Affiliations:** ^1^Department of Medico-Surgical Sciences and Biotechnologies, Sapienza University of Rome, Corso della Repubblica 79, 04100 Latina, Italy; ^2^Department of Cardiovascular Sciences, European Hospital, 00149 Rome, Italy; ^3^Department of Radiology, Copenhagen University Hospital, 2100 Herlev, Denmark; ^4^Division of Cardiology, Policlinico Casilino, 00169 Rome, Italy; ^5^Division of Cardiology, University of Turin, 10126 Turin, Italy; ^6^Department of Cardiovascular, Respiratory, Nephrologic and Geriatric Sciences, Sapienza University of Rome, 00185 Rome, Italy; ^7^VCU Pauley Heart Center, Virginia Commonwealth University, Richmond, VA 23298, USA; ^8^Department of Angiocardioneurology, IRCCS Neuromed, 86077 Pozzilli, Italy

## Abstract

Contrast-induced nephropathy is a common complication of iodinated contrast administration. Statins may reduce the risk of contrast-induced nephropathy, but data remain inconclusive. We summarized the evidence based on statins for the prevention of contrast-induced nephropathy with a network meta-analysis. Randomized trials focusing on statins were searched and pooled with random-effect odds ratios. A total of 14 trials (6,160 patients) were included, focusing on atorvastatin (high/low dose), rosuvastatin (high dose), simvastatin (high/low dose), and placebo or no statin therapy before contrast administration. The risk of contrast-induced nephropathy was reduced by atorvastatin high dose and rosuvastatin high dose, with no difference between these two agents. Results for atorvastatin low dose and simvastatin (high/low dose) in comparison to placebo were inconclusive. Atorvastatin and rosuvastatin administered at high doses and before iodinated contrast administration have a consistent and beneficial preventive effect on contrast-induced nephropathy and may actually halve its incidence.

## 1. Introduction

Iodinated contrast-induced nephropathy (CIN) is an important cause of hospital-acquired acute renal injury [1]. Patients with acute coronary syndrome (ACS) and/or baseline renal dysfunction, as well as those undergoing percutaneous coronary intervention (PCI), are at particularly high risk of CIN, which, when occurring, may be followed by persistent worsening of renal function [2, 3]. To date, there have been many studies focusing on the pathophysiology, epidemiology, risk prognostication, and prevention of CIN. Yet, the pathophysiology of CIN remains unclear but may be related to direct renal tubular toxicity, vasoconstriction, and high oxidative stress [4, 5].

Statins are primarily used in cardiovascular medicine for their lipid-lowering effects. However, they have recently been shown to possess remarkable pleiotropic effects such as improving endothelial function as well as decreasing oxidative stress and inflammation [6]. In the context of cardiovascular disease, nitric oxide-derived oxidant species that promote atherogenesis are suppressed by statins [7]. Therefore, statins are considered as promising candidate agents for the prevention of CIN.

A number of studies have shown that statins may have a protective effect on CIN, but there remains uncertainty regarding time and way of administration of statins and it is not clear whether a specific statin is better than the others. Pairwise meta-analyses combine the results of homogeneous studies conducted on the same topic, whereas network meta-analyses evaluate simultaneously both direct and indirect comparisons across trials sharing one or more common comparators [21–23]. The purpose of our work was thus to perform a systematic review including both pairwise and network meta-analysis in order to evaluate more accurately the effect of statins compared to placebo or standard therapies for the prevention of CIN.

## 2. Methods

### 2.1. Design

The present review was conducted in keeping with the current guidelines from the Preferred Reporting Items for Systematic reviews and Meta-Analyses (PRISMA) group and from the Cochrane Collaboration. All reviewing activities were conducted by two independent reviewers (MP, GBZ), with divergences solved after consensus.

### 2.2. Search

MEDLINE/PubMed was searched for suitable studies according to Biondi-Zoccai et al., with the following substring: [24] (statin∗ OR atorvastatin OR rosuvastatin OR simvastatin OR lovastatin OR fluvastatin) AND (contrast AND (nephropathy OR ((renal OR kidney) AND (injury OR damage)))). In addition, the Cochrane Library, Google Scholar, and Scopus were searched for additional studies. No language restriction was enforced in order to minimize the risk of publication bias, actually leading to the inclusion of a study published only in Chinese [8]. Searches were last updated on November 14, 2013, with the exception of the MEDLINE/PubMed query, which was last performed on July 15, 2014.

### 2.3. Selection

Citations were initially screened at the title/abstract level. If potentially pertinent, they were then retrieved as full texts. Studies were included if randomized, allocating one or more groups to statin therapy and reporting dichotomous outcomes relevant to the assessment of CIN. Accordingly, studies were excluded if not randomized, duplicated, focusing on nonstatin strategies, or lacking suitable outcome details.

### 2.4. Abstraction and Validity

Study, patient, and procedural features were systematically extracted, with a particular focus on treatment strategy, type and volume of contrast, and CIN-related outcomes. The primary endpoint was the occurrence, at the longest in-hospital follow-up, of CIN, defined as a relative increase in serum creatinine ≥25% or an absolute increase in serum creatinine ≥0.5 mg/dL (44 *μ*mol/L), typically 48–72 hours after the index procedure. In addition, the following endpoints were also collected at the longest in-hospital follow-up: absolute increase in serum creatinine ≥0.5 mg/dL (44 *μ*mol/L); relative increase in serum creatinine ≥25%. Conversely, the longest available clinical follow-up (thus well beyond hospitalization, when available) was exploited to abstract data on all-cause death and need for dialysis or renal replacement therapy. Study validity was appraised in keeping with ongoing recommendations distinguishing several sources of bias typical of randomized trials [23].

### 2.5. Analysis

Categorical variables are described as* n* (%) and continuous variables as median. Pairwise meta-analysis was performed within a frequentist framework, computing odds ratios (OR, 95% confidence intervals) by means of a Mantel-Haenszel random-effect method [21]. Network meta-analysis was performed within a Bayesian framework computing odds ratios (95% credible intervals) and probability of being best of any given treatment (Pbest) with a fixed-effect hierarchical model, sampling posterior probabilities by means of Markov chain Monte Carlo (MCMC) methods with Gibbs sampling from 150,000 iterations obtained after a 50,000-iteration training phase [22, 23]. Convergence was appraised graphically according to Gelman and Rubin, and noninformative priors were used throughout. A fixed-effect model was used for network meta-analysis based on the star-shaped evidence network, which is typically analyzed with such an approach, and after having confirmed that model fit was not different from a random-effect and a fixed-effect model computing values for deviance information criterion (DIC). Pairwise heterogeneity was appraised with chi-squared tests, whereas network inconsistency was evaluated comparing the results stemming from consistent and inconsistent models [21, 23]. Small study effects (e.g., publication bias) were explored by visual inspection of funnel plots. Computations were performed with RevMan and WinBUGS [23].

## 3. Results

**Figure 1:**
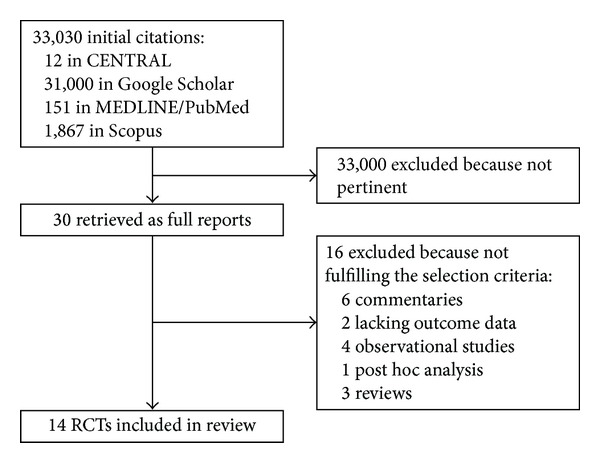
Review profile.

**Figure 2:**
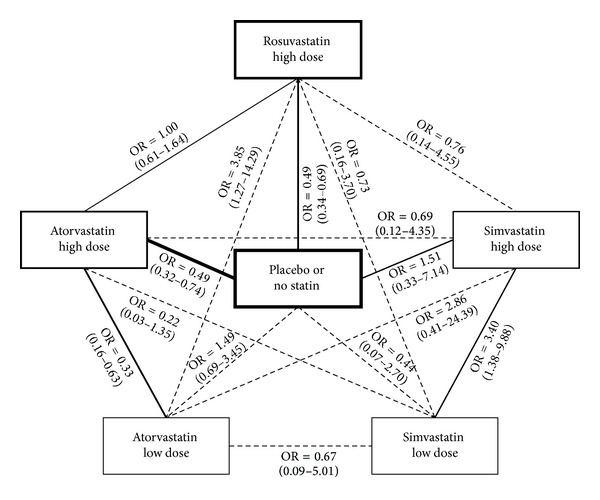
Evidence network. Continuous lines represent head-to-head randomized comparisons (the thickness of the line corresponding to the number of trials) with both direct and indirect effect estimates, whereas dashed lines represent only indirect effect estimates. The thickness of the rectangles corresponds to the patients receiving a specific treatment.

**Figure 3:**
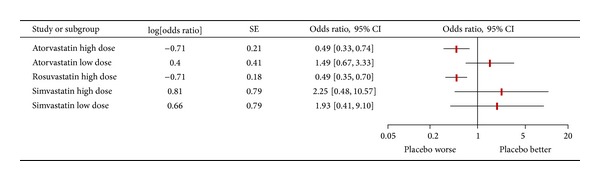
Forest plot summarizing risk estimates stemming from network meta-analysis comparing different statin regimens for the risk of contrast-induced nephropathy. OR = odds ratio; CI = confidence interval; SE = standard error.

From an initial set of 33,030 citations retrieved from multiple databases, a total of 30 articles were analyzed as full reports according to our explicit selection criteria, finally yielding 14 studies eligible for inclusion in our review (Figure 1). These trials included a total of 6,160 patients, according to the following evidence network (Figures 2 and 3; Tables 1, 2, 3, and 4): 4 studies (706 subjects) compared atorvastatin high dose with atorvastatin low dose [8, 10, 14, 20], 5 studies (1246 subjects) compared atorvastatin high dose with placebo or standard therapy without statins [13, 15–18], 1 study (192 subjects) compared atorvastatin high dose with rosuvastatin high dose [11], 2 studies (3541 subjects) compared rosuvastatin high dose with placebo or standard therapy without statins [4, 12], 1 study (247 subjects) compared simvastatin high dose with placebo or standard therapy without statins [9], and 1 study (228 subjects) compared simvastatin high dose with simvastatin low dose [19].

Studies were clinically diverse, being conducted in Asia and Europe, and included patients with diabetes mellitus (median prevalence 23%) or mild to moderate chronic kidney disease (median baseline serum creatinine 1.0 mg/dL 1 (88 *μ*mol/L)). Several types of iodinated contrast media were used, including iobitridol, iodixanol, iohexol, iopamidol, and iopromide (median volume 161 mL). Study quality was adequate in most cases, but satisfactory details on randomization procedures and thorough double blinding were present only for 5 trials [9, 13, 16–18].

Pairwise meta-analysis for CIN was first conducted to explore for overall clinical effects (online Figures 1–13 in Supplementary Material available online at http://dx.doi.org/10.1155/2014/213239). These computations showed that statins at high dose were associated with a reduced risk of CIN (OR = 0.47 (95% confidence interval 0.37–0.60),* P* for effect <0.001,* P* for heterogeneity = 0.59, and I-squared = 0%), of an absolute increase in serum creatinine ≥0.5 mg/dL (OR = 0.57 (0.32–1.02),* P* for effect = 0.06,* P* for heterogeneity = 0.26, and I-squared = 25%), and of a relative increase in serum creatinine ≥25% (OR = 0.35 (0.17–0.69),* P* for effect = 0.003,* P* for heterogeneity = 0.57, and I-squared = 0%). In addition, statins at high dose were associated with trends toward reduced risks of in-hospital dialysis (OR = 0.27 (0.07–1.09),* P* for effect = 0.07,* P* for heterogeneity = 1.0, and I-squared = 0%) and death at a median follow-up of 3 months (OR = 0.80 (0.31–2.10),* P* for effect = 0.65,* P* for heterogeneity = 0.61, and I-squared = 0%).

Subsequently, network meta-analysis was performed for the risk of CIN, distinguishing atorvastatin high dose, atorvastatin low dose, rosuvastatin high dose, simvastatin high dose, and simvastatin low dose. These computations showed that atorvastatin high dose was the regimen which was most likely to be beneficial (OR versus placebo = 0.49 (95% credible interval 0.32–0.74), Pbest = 34%), followed by rosuvastatin high dose (OR versus placebo = 0.49 (0.34–0.69), Pbest = 34%). Head-to-head comparison of atorvastatin high dose versus rosuvastatin high dose suggested a very similar effect (OR = 1.00 (0.61–1.64)). Data for simvastatin high dose were apparently favorable, but inconclusive (OR versus placebo = 0.66 (0.14–3.04), Pbest = 32%), whereas data for atorvastatin low dose and simvastatin low dose were clearly not in favor of clinical use to prevent CIN (resp. OR versus placebo = 1.49 (0.69–3.45), Pbest < 0.1%, and OR versus placebo = 2.25 (0.37–14.61), Pbest = 0.2%). Additional network analyses for the other CIN endpoints, dialysis, or death were not possible for the paucity of reported events.

Consistency of pairwise and network analyses was satisfactory, in light of the low I-squared estimates and the similar results yielded by consistent, inconsistent, fixed-effect, and random-effect Bayesian models. Even sensitivity analysis excluding the largest study (Han et al.) [4] confirmed in direction and magnitude of effects the overall analysis (Table 5). Finally, no clear evidence of small study effects was found at funnel plot inspection (online Figures 3, 6, 9, 11, and  13).

## 4. Discussion

This review, the first in the literature exploiting the totality of the evidence base with the novel mixed treatment comparison approach for comparative effectiveness analysis, confirms that statins at high dose reduce the risk of CIN in patients undergoing coronary angiography or revascularization with current iodinated contrast media, with potentially beneficial effects also on the risk of in-hospital dialysis. Moreover, network meta-analysis suggests that atorvastatin and rosuvastatin at high dose represent the first choice regimens in order to achieve a consistent and beneficial preventive effect on CIN and may actually halve its incidence.

Invasive angiography exploiting iodinated contrast media may be complicated in several cases by CIN, with a negative impact on prognosis, hospital stay, and costs [25]. Accordingly, means to prevent it may yield important and clinically relevant benefits. Yet, its pathophysiology remains unclear. It may be due to direct toxicity on the renal tubular epithelium, oxidative stress, ischemic injury, and renal tubular obstruction, as any change in nitric oxide, prostaglandins, endothelin, and adenosine in the vasoregulation pathway could contribute to worsen medullary ischemia [26]. Many preventive strategies and treatments have been proposed for CIN, although only few are with a demonstrated clinical efficacy [25, 27, 28].

In addition to their impact on cholesterol, statins are known to have multiple nonlipid lowering (i.e., pleiotropic) effects, which include several mechanisms involving inflammation responses, endothelial function, oxidative stress, and apoptotic pathways [17]. Preventing contrast-induced renal cell apoptosis seems to play an important role in statins effects on CIN. Because inflammation is unquestionably involved in the pathogenesis of kidney injury, its modulation could be part of the mechanism expounding the reduced incidence of CIN after treatment with statins. The antioxidant effect of statins in addition to other antioxidant compounds (sodium bicarbonate solution and N-acetylcysteine) seems to reduce the occurrence of CIN just through scavenging reactive oxygen species (ROS) [14]. Khanal et al. firstly reported, in a retrospective study that evaluated a large cohort of patients who underwent PCI, that patients on chronic statin therapy before the procedure had a significantly lower incidence of CIN [29]. Thereafter, several prospective nonrandomized or randomized placebo controlled trials suggested that a short-term regimen of high-dose statins before and after contrast exposure, in addition to standard measures for preventing kidney deterioration, might decrease the incidence of CIN. Nevertheless, the type and the dose threshold of statins to reduce the risk of CIN remained uncertain.

Our meta-analysis represents the first work to exploit network meta-analytic methods to compare different statins in their CIN preventive effects. It shows that atorvastatin and rosuvastatin, when administered at high dose, represent the most effective preventive strategy. Accordingly, the present work provides important findings in support of the favorable risk-benefit balance of statins to prevent CIN, as offering patients a short-term yet high-dose statin regimen before administration of iodinated contrast appears as a remarkably safe, simple, and effective intervention.

Focusing on current pharmacopeia, all statins are administered in active form except lovastatin and simvastatin which are given as a prodrug and converted in vivo into the liver. More of 90% of statins are bound to plasma proteins, but rosuvastatin has a lower rate of link (88%). This is due to the hydrophilicity features of this molecule limiting the need for its transport in the blood through albumin. Hydrophilic statins encompass acute pleiotropic effects. Moreover, they do not undergo cytochrome P-450 3A4 metabolism in the liver. These features may explain, at least in part, potential differences between statins in CIN prevention. In addition, as CIN is a paradigm of mutual hypoxic and toxic renal parenchymal injury mediated, to large extent, by an increased production of mediators of inflammation and ROS and clinical as well as experimental findings clearly illustrate that hypoxia and enhanced ROS formation within the kidney following contrast media administration play a critical role in the development of CIN [30], we speculate that atorvastatin and rosuvastatin may be more effective than other statins probably due to their higher anti-inflammation and antioxidant features.

This work has several limitations, including those typical of systematic reviews, pairwise meta-analyses and mixed treatment comparisons [21, 23]. More poignantly to the current findings, we exploited an evidence network with a prevalent star shape. In addition, we relied on surrogate outcomes for renal function, which have been called into question for being overly sensitive and subject to substantial between- and within-individual variability. In particular, relative increases in serum creatinine ≥25% may occur in many patients irrespectively of the amount of administered contrast. In addition, no study focused on noncoronary contrast media administration, and thus the implications of our results for other imaging procedures (e.g., computed tomography) are open to individual interpretation. Finally, due to the lack of patient-level data, key moderators on the risk of CIN, such as means of contrast administration (intravenous versus intra-arterial), volume of contrast, type of contrast [25], and baseline use of statins, could not be appraised quantitatively.

## 5. Conclusion

Atorvastatin and rosuvastatin, when administered at high doses and before iodinated contrast administration, have a consistent and beneficial preventive effect on CIN and may actually halve its incidence.

## Supplementary Material

The Supplementary Material includes complementary information for the pairwise and sensitivity analyses.

## Figures and Tables

**Table 1:** Included studies.

First author	Year	Journal	Patients	Multicenter setting	Location	Follow-up (days)	Selection criteria
Cao [8]	2012	Nan Fang Yi Ke Da Xue Xue Bao	180	No	China	3	ACS undergoing coronary angiography or PCI without renal failure

Han [4]	2013	Journal of the American College of Cardiology	2998	Yes	China	30	Type 2 DM and stage 2-3 CKD

Jo [9]	2008	American Heart Journal	247	Yes	Korea	180	CKD, statin naive, undergoing coronary angiography

Jo [10]	2014	Journal of Cardiovascular Medicine	218	Yes	Korea	180	STEMI undergoing emergency PCI irrespective of renal dysfunction

Kaya [11]	2013	Acta Cardiologica	192	No	Turkey	2	STEMI undergoing emergency PCI without renal dysfunction

Leoncini [12]	2014	Journal of the American College of Cardiology	543	No	Italy	180	NSTE-ACS, statin naive, selected for early invasive strategy, without acute or end-stage renal failure

Li [13]	2012	Cardiology	161	No	China	30	STEMI undergoing emergency PCI without renal dysfunction

Li [14]	2014	Scientific World Journal	208	No	China	1	Patients undergoing coronary angiography or angioplasty

$\ddot{\text{O}}$zhan [15]	2010	Angiology	130	No	Turkey	2	Patients undergoing coronary angiography without renal insufficiency (serum creatinine <1.5 mg/dL)

Patti [16]	2011	American Journal of Cardiology	241	Yes	Italy	2	NSTE-ACS, statin naive, receiving early PCI

Quintavalle [17]	2012	Circulation	410	Yes	Italy	365	CKD, statin naive, scheduled for elective coronary angiography or PCI

Toso [18]	2010	American Journal of Cardiology	304	No	Italy	30	CKD, statin naive, without end-stage renal failure requiring dialysis

Xinwei [19]	2009	American Journal of Cardiology	228	No	China	7	ACS undergoing PCI

Zhou [20]	2009	Zhonghua Xin Xue Guan Bing Za Zhi	100	No	China	3	Coronary angiography or PCI

ACS = acute coronary syndrome; CKD = chronic kidney disease; DM = diabetes mellitus; NSTE-ACS = non-ST-elevation ACS; PCI = percutaneous coronary intervention; STEMI = ST-elevation myocardial infarction.

**Table 2:** Patient and procedural features.

First author	Year	Experimental therapy	Control therapy	Contrast type	Median contrast volume	Age (years)	Diabetes mellitus	Baseline serum creatinine (mg/dL)
Cao [8]	2012	Atorvastatin 40 mg/day started 3 days before angioplasty followed by atorvastatin 20 mg/day	Atorvastatin 20 mg/day	NA	161	63	20%	NA

Han [4]	2013	Rosuvastatin 10 mg/day from 2 days before to 3 days after contrast	No statin	Iodixanol	115	61	100%	1.1

Jo [9]	2008	Simvastatin 40 + 40 mg before angiography followed by simvastatin 40 + 40 mg afterwards	Placebo	Iodixanol	182	65	26%	1.2

Jo [10]	2014	Atorvastatin 80 mg before angiography followed by atorvastatin 80 mg/day for 5 days and then 10 mg/day	Atorvastatin 10 mg/day	NA	NA	60	26%	1.1

Kaya [11]	2013	Atorvastatin 80 mg before angiography	Rosuvastatin 40 mg before angiography	Iopromide	153	63	20%	0.9

Leoncini [12]	2014	Rosuvastatin 40 mg at admission followed by 20 mg/day	No statin	Iodixanol	261	66	21%	1.0

Li [13]	2012	Atorvastatin 80 mg at admission followed by atorvastatin 40 mg after angiography	Placebo	Iopromide	102	66	28%	0.9

Li [14]	2014	Atorvastatin 40 mg before angiography, followed by atorvastatin 40 mg/day	Atorvastatin 20 mg/day	Iopamidol	186	61	24%	0.9

$\ddot{\text{O}}$zhan [15]	2010	Atorvastatin 80 mg before angiography, followed by atorvastatin 80 mg 48 hours after contrast administration	No statin	Iopamidol	95	55	16%	0.8

Patti [16]	2011	Atorvastatin 80 mg 12 hrs before angiography; further 40 mg preprocedure (2 hrs before), followed by atorvastatin 40 mg/day	Placebo	Iobitridol	211	66	27%	1.0

Quintavalle [17]	2012	Atorvastatin 80 mg (within 24 hrs before contrast exposure), followed by atorvastatin 20 mg/day	No statin	Iodixanol	180	70	41%	1.3

Toso [18]	2010	Atorvastatin 80 mg 48 hours before and 48 hours after contrast administration	Placebo	Iodixanol	157	75	21%	1.2

Xinwei [19]	2009	Simvastatin 80 mg from admission to day before PCI, followed by simvastatin 20 mg/day	Simvastatin 20 mg/day	Iodixanol or iohexol	233	65	21%	0.8

Zhou [20]	2009	Atorvastatin 80 mg/day before the procedure, 10 mg/day for 6 days after procedure	Atorvastatin 10 mg/day for 7 days	Iopamidol	116	61	20%	1.1

NA = not available or applicable.

**Table 3:** Internal validity of included studies.

First author	Year	Inadequate allocation sequence generation?	Inadequate allocation concealment?	Inadequate blinding?	Incomplete outcome data?	Selective outcome reporting?	Risk of other bias?
Cao [8]	2012	Unclear	Unclear	Unclear	No	Yes (lack of some individual CIN data)	No
Han [4]	2013	No	No	Yes (open label)	No	Yes (lack of some individual CIN data)	No
Jo [9]	2008	No	No	No	No	Yes (lack of some individual CIN data)	No
Jo [10]	2014	No	Unclear	Yes (open label)	No	Yes (lack of some individual CIN data)	No
Kaya [11]	2013	Unclear	Unclear	Yes (open label)	No	Yes (lack of some individual CIN data)	No
Leoncini [12]	2014	No	Yes (open-label list)	Yes (open label)	No	Yes (lack of some individual CIN data)	No
Li [13]	2012	No	No	No	No	Yes (lack of some individual CIN data)	No
Li [14]	2014	Unclear	Unclear	Yes (open label)	No	Yes (lack of some individual CIN data)	No
$\ddot{\text{O}}$zhan [15]	2010	Unclear	Unclear	Unclear	No	Yes (lack of some individual CIN data)	No
Patti [16]	2011	No	No	No	No	Yes (lack of some individual CIN data)	No
Quintavalle [17]	2012	No	No	No	No	Yes (lack of some individual CIN data)	No
Toso [18]	2010	No	No	No	No	Yes (lack of some individual CIN data)	No
Xinwei [19]	2009	No	Yes (open-label study)	Yes (open label)	No	Yes (lack of some individual CIN data)	No
Zhou [20]	2009	Unclear	Unclear	Unclear	No	Yes (lack of some individual CIN data)	No

CIN = contrast-induced nephropathy.

**Table 4:** Effect estimates for the risk of contrast-induced nephropathy. Results are reported as probability of being treatment (Pbest) and odds ratios (OR) with 95% credible intervals of a given row item versus a corresponding column item. Thus, OR < 1 indicates lower risk in the corresponding row item and OR > 1 indicates lower risk in the corresponding column item.

Treatment	Atorvastatin high dose	Atorvastatin low dose	Rosuvastatin high dose	Simvastatin high dose	Simvastatin low dose	Placebo or no statin
Atorvastatin high dose	Pbest = 34%	**OR = 0.33 (0.16–0.63)**	OR = 1.00 (0.61–1.64)	OR = 0.69 (0.12–4.35)	OR = 0.22 (0.03–1.35)	**OR = 0.49 (0.32–0.74)**
Atorvastatin low dose	**OR = 3.05 (1.59–6.21)**	Pbest < 0.1%	**OR = 3.85 (1.27–14.29)**	OR = 2.86 (0.41–24.39)	OR = 0.67 (0.09–5.01)	OR = 1.49 (0.69–3.45)
Rosuvastatin high dose	OR = 1.00 (0.61–1.63)	**OR = 0.26 (0.07–0.79)**	Pbest = 34%	OR = 0.76 (0.14–4.55)	OR = 0.73 (0.16–3.70)	**OR = 0.49 (0.34–0.69)**
Simvastatin high dose	OR = 1.44 (0.23–8.11)	OR = 0.35 (0.04–2.42)	OR = 1.32 (0.22–7.13)	Pbest = 32%	**OR = 0.29 (0.10–0.72)**	OR = 0.66 (0.14–3.04)
Simvastatin low dose	OR = 4.61 (0.74–30.37)	OR = 1.50 (0.20–10.86)	OR = 1.37 (0.27–6.42)	**OR = 3.40 (1.38–9.88)**	Pbest = 0.2%	OR = 2.25 (0.37–14.61)
Placebo or no statin	**OR = 2.05 (1.35–3.09)**	OR = 0.67 (0.29–1.44)	**OR = 2.04 (1.44–2.94) **	OR = 1.51 (0.33–7.14)	OR = 0.44 (0.07–2.70)	Pbest = 0

**Table 5:** Effect estimates for the risk of contrast-induced nephropathy excluding the study by Han et al. [4]. Results are reported as probability of being best treatment (Pbest) and odds ratios (OR) with 95% credible intervals of a given row item versus a corresponding column item. Thus, OR < 1 indicates lower risk in the corresponding row item and OR > 1 indicates lower risk in the corresponding column item.

Treatment	Atorvastatin high dose	Atorvastatin low dose	Rosuvastatin high dose	Simvastatin high dose	Simvastatin low dose	Placebo or no statin
Atorvastatin high dose	Pbest = 20%	**OR = 0.32 (0.15–0.65)**	OR = 1.22 (0.66–2.33)	OR = 0.65 (0.15–3.85)	OR = 0.19 (0.03–1.37)	**OR = 0.47 (0.31–0.70)**
Atorvastatin low dose	**OR = 3.12 (1.53–6.73)**	Pbest < 0.1%	**OR = 3.85 (1.47–11.09)**	OR = 2.08 (0.36–14.08)	OR = 0.60 (0.08–4.98)	OR = 1.45 (0.62–3.45)
Rosuvastatin high dose	OR = 0.82 (0.43–1.51)	**OR = 0.26 (0.10–0.68)**	Pbest = 34%	OR = 0.53 (0.12–3.23)	OR = 0.16 (0.02–1.22)	**OR = 0.38 (0.22–0.65)**
Simvastatin high dose	OR = 1.54 (0.26–6.87)	OR = 0.48 (0.07–2.77)	OR = 1.90 (0.31–8.56)	Pbest = 20%	**OR = 0.29 (0.10–0.78)**	OR = 0.72 (0.13–3.06)
Simvastatin low dose	OR = 5.24 (0.73–32.12)	OR = 1.67 (0.20–12.66)	OR = 6.46 (0.82–41.28)	**OR = 3.42 (1.28–10.03)**	Pbest = 0.2%	OR = 2.46 (0.35–14.17)
Placebo or no statin	**OR = 2.15 (1.42–3.28)**	OR = 0.69 (0.29–1.61)	**OR = 2.63 (1.54–4.55)**	OR = 1.39 (0.33–7.69)	OR = 0.41 (0.07–2.86)	Pbest = 0
