# Supplementary material for: A Network Meta-Analysis on Randomized Trials Focusing on the Preventive Effect of Statins on Contrast-Induced Nephropathy
Source: Biomed Res Int. 2014 Sep 7;2014:213239. doi: 10.1155/2014/213239 (PMC4170696; doi:10.1155/2014/213239)
Supplement: Supplementary file 1 — The Supplementary Material includes complementary information for the pairwise and sensitivity analyses. [file 213239.f1.zip › supplementary material/1056707.pptx]

## Slide 1
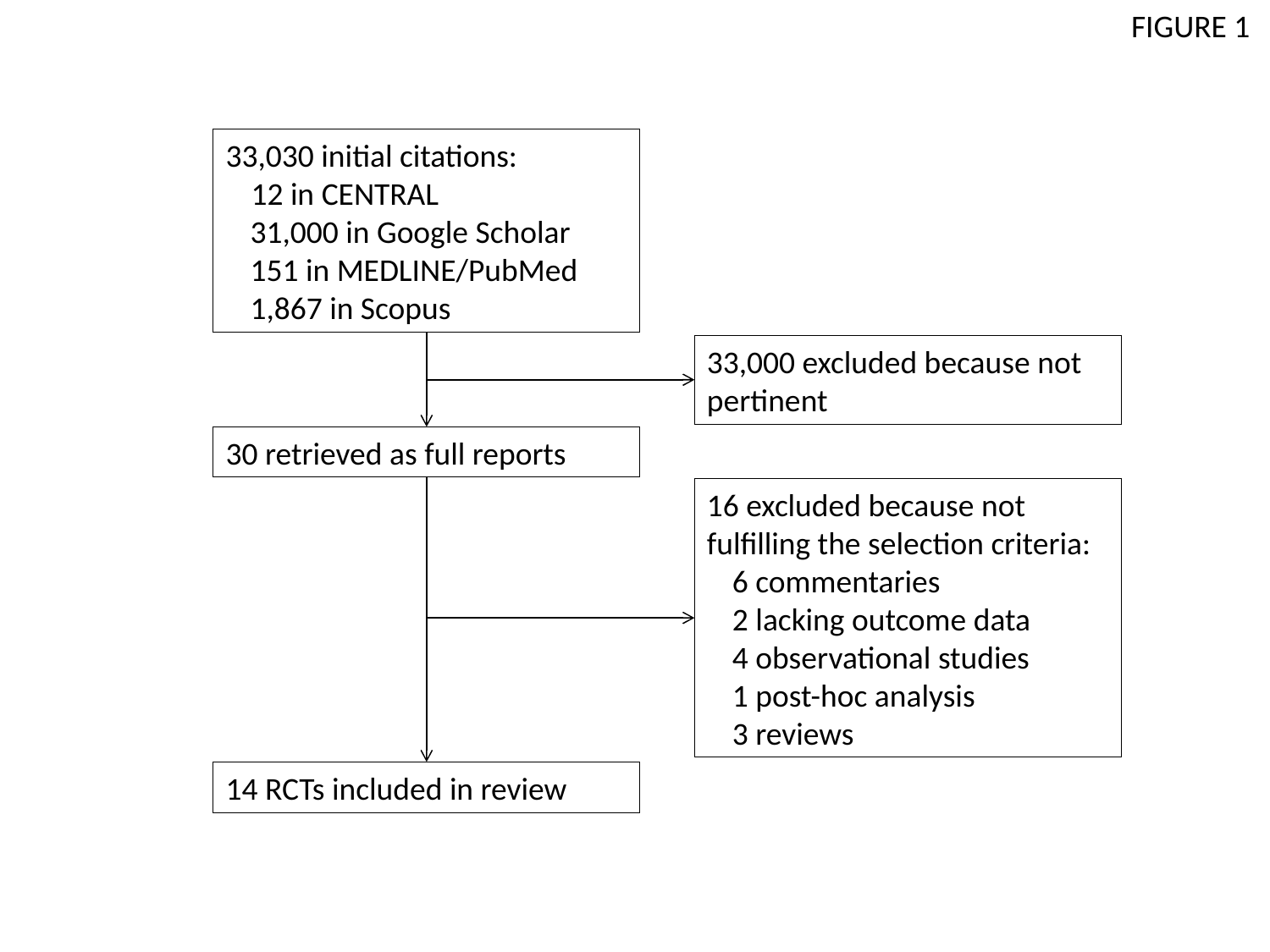

FIGURE 1
33,030 initial citations:
12 in CENTRAL
31,000 in Google Scholar
151 in MEDLINE/PubMed
1,867 in Scopus
33,000 excluded because not pertinent
30 retrieved as full reports
16 excluded because not fulfilling the selection criteria:
6 commentaries
2 lacking outcome data
4 observational studies
1 post-hoc analysis
3 reviews
14 RCTs included in review

## Slide 2
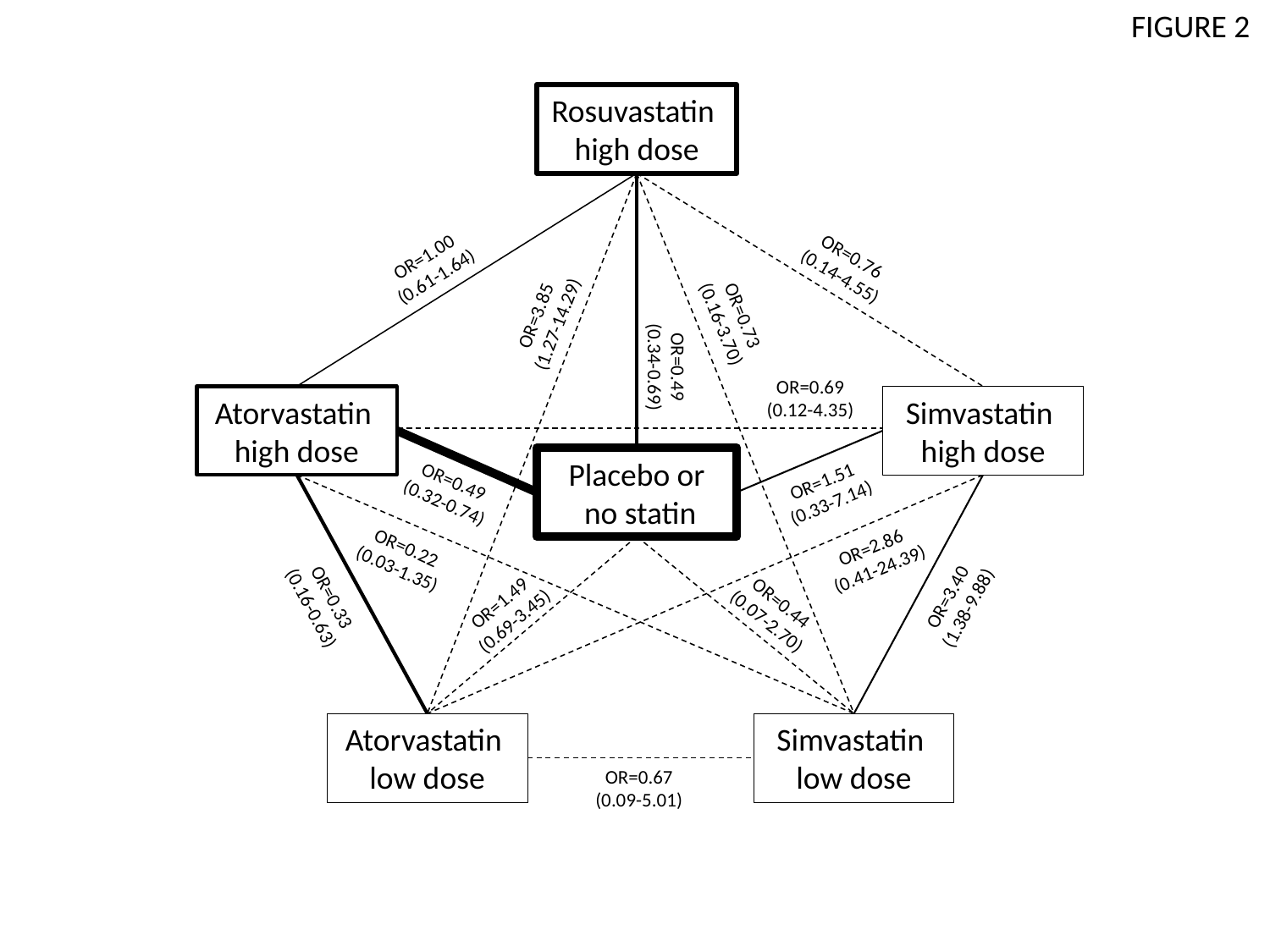

FIGURE 2
Rosuvastatin
high dose
OR=1.00 (0.61-1.64)
OR=0.76 (0.14-4.55)
OR=3.85 (1.27-14.29)
OR=0.73 (0.16-3.70)
OR=0.49 (0.34-0.69)
OR=0.69 (0.12-4.35)
Atorvastatin
high dose
Simvastatin
high dose
Placebo or
 no statin
OR=0.49 (0.32-0.74)
OR=1.51 (0.33-7.14)
OR=0.22 (0.03-1.35)
OR=2.86 (0.41-24.39)
OR=0.33 (0.16-0.63)
OR=3.40 (1.38-9.88)
OR=1.49 (0.69-3.45)
OR=0.44 (0.07-2.70)
Atorvastatin
low dose
Simvastatin
low dose
OR=0.67 (0.09-5.01)

## Slide 3
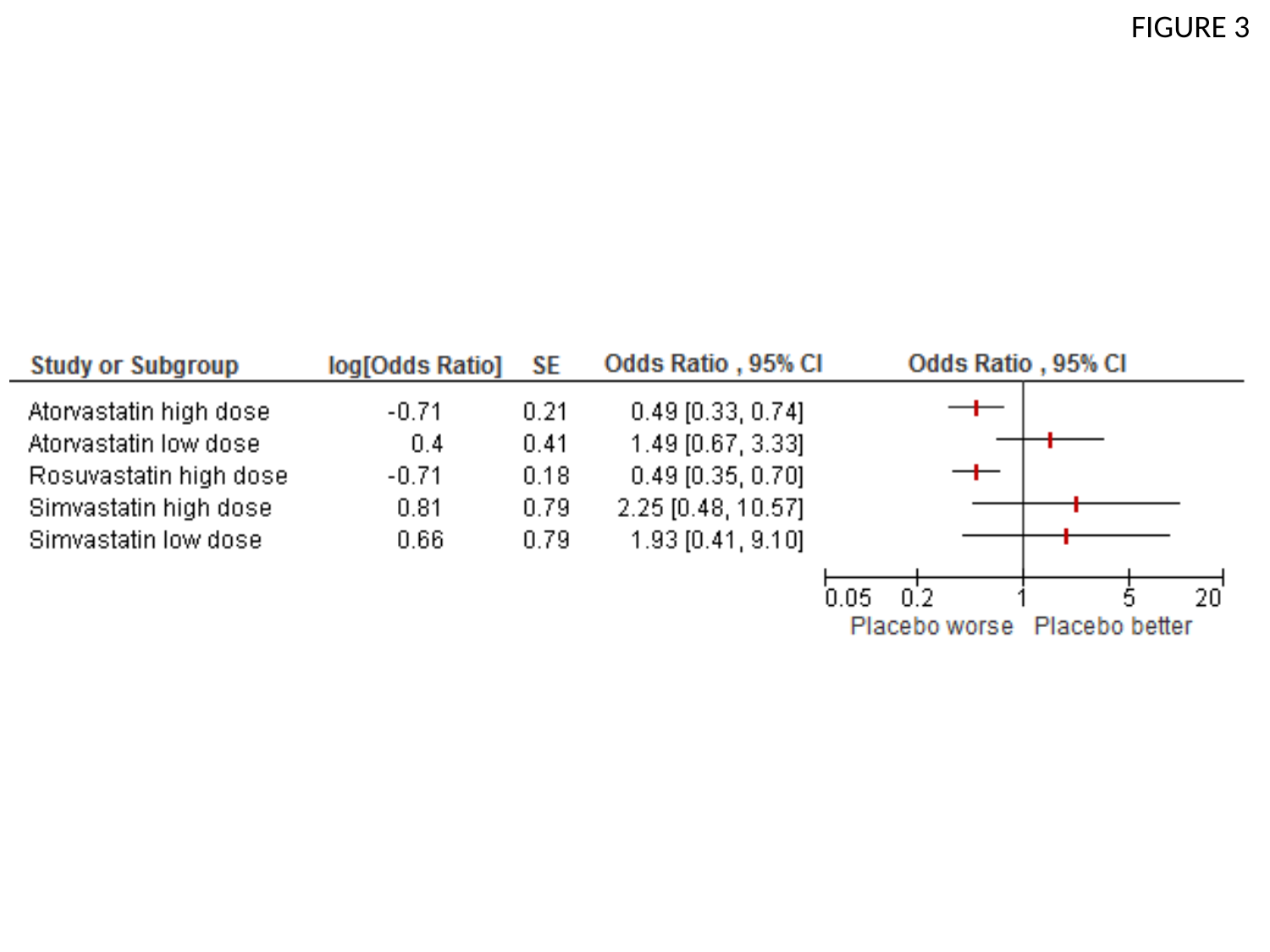

FIGURE 3
