# Supplementary figures and images for: A Network Meta-Analysis on Randomized Trials Focusing on the Preventive Effect of Statins on Contrast-Induced Nephropathy
Source: Biomed Res Int. 2014 Sep 7;2014:213239. doi: 10.1155/2014/213239 (PMC4170696; doi:10.1155/2014/213239)

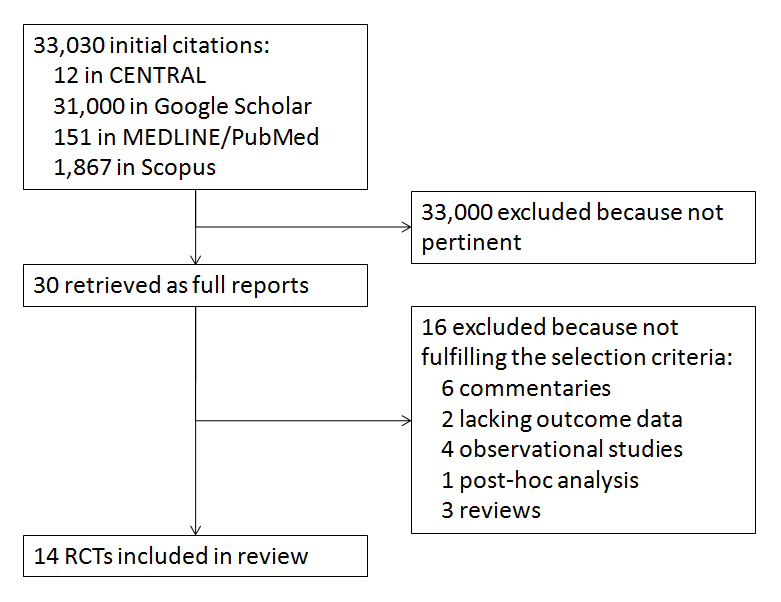

Supplement: Supplementary file 1 — The Supplementary Material includes complementary information for the pairwise and sensitivity analyses. [file 213239.f1.zip › supplementary material/1048285.docx]

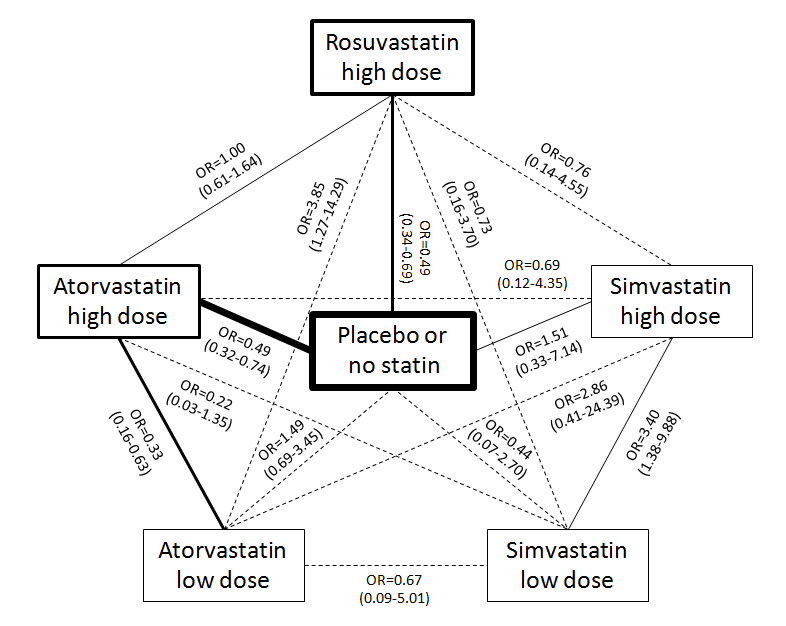

Supplement: Supplementary file 1 — The Supplementary Material includes complementary information for the pairwise and sensitivity analyses. [file 213239.f1.zip › supplementary material/1048286.docx]

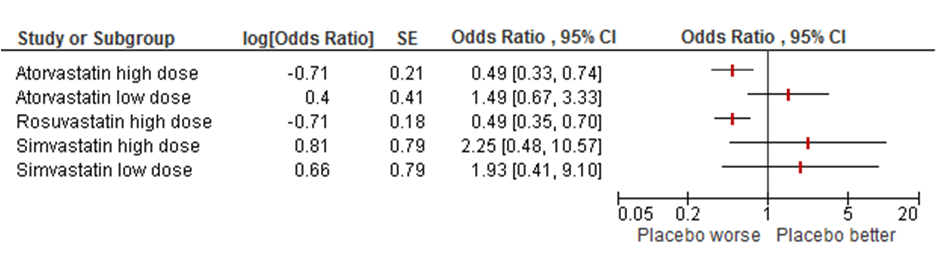

Supplement: Supplementary file 1 — The Supplementary Material includes complementary information for the pairwise and sensitivity analyses. [file 213239.f1.zip › supplementary material/1048287.docx]
