# Supplementary material for: A Network Meta-Analysis on Randomized Trials Focusing on the Preventive Effect of Statins on Contrast-Induced Nephropathy
Source: Biomed Res Int. 2014 Sep 7;2014:213239. doi: 10.1155/2014/213239 (PMC4170696; doi:10.1155/2014/213239)
Supplement: Supplementary file 1 — The Supplementary Material includes complementary information for the pairwise and sensitivity analyses. [file 213239.f1.zip › supplementary material/1048283.doc]

**A network meta-analysis on randomized trials focusing on the preventive effect of statins on contrast-induced nephropathy**

Mariangela Peruzzi,^1^ Leonardo De Luca,^2^ Henrik S. Thomsen,^3^ Enrico Romagnoli,^4^ Fabrizio D’Ascenzo,^5^ Massimo Mancone,^6^ Gennaro Sardella,^6^ Luigi Lucisano,^6^ Antonio Abbate, MD, PhD,^7^ Giacomo Frati,^1,8^ Giuseppe Biondi-Zoccai^1,7^

^1^Department of Medico-Surgical Sciences and Biotechnologies, Sapienza University of Rome, Latina Italy; ^2^Department of Cardiovascular Sciences, European Hospital, Rome, Italy; ^3^Department of Radiology, Copenhagen University Hospital, Herlev, Denmark; ^4^Division of Cardiology, Policlinico Casilino, Rome, Italy ; ^5^Division of Cardiology, University of Turin, Turin, Italy; ^6^Department of Cardiovascular, Respiratory, Nephrologic and Geriatric Sciences, Sapienza University of Rome, Rome, Italy; ^7^VCU Pauley Heart Center, Virginia Commonwealth University, Richmond, VA, USA; ^8^Department of AngioCardioNeurology, IRCCS Neuromed, Pozzilli, Italy

M Peruzzi: mariangela.peruzzi@uniroma1.it; L De Luca: leo.deluca@libero.it; H Thomsen: Henrik.Thomsen@regionh.dk; E Romagnoli: enromagnoli@gmail.com; F D’Ascenzo: fabrizio.dascenzo@gmail.com; M Mancone: massimo.mancone@uniroma1.it; G Sardella: gennaro.sardella@uniroma.it; L Lucisano: luigi.lucisano@uniroma1.it; A Abbate: abbatea@yahoo.com; G Frati: giacomo.frati@uniroma1.it; G Biondi-Zoccai: giuseppe.biondizoccai@uniroma1.it

**Running title**: Statins for contrast-induced nephropathy	**Text word count**: 4,983

**Corresponding author**: Dr. Mariangela Peruzzi, Department of Medico-Surgical Sciences and Biotechnologies, Sapienza University of Rome, Corso della Repubblica 79, 04100 Latina, Italy. Phone: +39 0773 1757245. Fax: +39 0773 1757254. Email: mariangela.peruzzi@uniroma1.it

**ABSTRACT**

**Background:** Contrast-induced nephropathy (CIN) is a common complication of iodinated contrast administration. Statins may reduce the risk of CIN, but data remain inconclusive. We summarized the evidence base on statins for the prevention of CIN with a network meta-analysis.

**Methods:** Randomized trials focusing on statins for the prevention of CIN were searched. Random-effect odds ratios (OR) and probability of being the best treatment (Pbest) were computed for the in-hospital rate of CIN, defined as an absolute increase of at least 0.5 mg/dL (44 µmol/L) or a relative increase of at least 25% in serum creatinine.

**Results:** A total of 14 trials (6,160 patients) were included, focusing on atorvastatin (high/low dose), rosuvastatin (high dose), simvastatin (high/low dose) and placebo or no statin therapy before contrast administration. The risk of CIN was reduced by atorvastatin high dose (OR versus placebo=0.49 [0.32-0.74]; Pbest=34%) and rosuvastatin high dose (OR versus placebo=0.49 [0.34-0.69]; Pbest=34%), with no difference between these two agents (OR of atorvastatin high dose versus rosuvastatin high dose=1.00 [0.61-1.64]). Results for atorvastatin low dose and simvastatin (high/low dose) in comparison to placebo were inconclusive.

**Conclusions:** Atorvastatin and rosuvastatin administered at high doses and before iodinated contrast administration have a consistent and beneficial preventive effect on CIN and may actually halve its incidence.

**KEY-WORDS**

Contrast-induced nephropathy; Coronary artery disease; Meta-analysis; Network meta-analysis; Statins

**INTRODUCTION**

Iodinated contrast-induced nephropathy (CIN) is an important cause of hospital-acquired acute renal injury [1]. Patients with acute coronary syndrome (ACS) and/or baseline renal dysfunction, as well as those undergoing percutaneous coronary intervention (PCI), are at particularly high risk of CIN, which, when occurring, may be followed by persistent worsening of renal function [2, 3]. To date, there have been many studies focusing on the pathophysiology, epidemiology, risk-prognostication and prevention of CIN. Yet, the pathophysiology of CIN remains unclear, but may be related to direct renal tubular toxicity, vasoconstriction and high oxidative stress [4, 5].

Statins are primarily used in cardiovascular medicine for their lipid-lowering effects. However, they have recently been shown to possess remarkable pleiotropic effects such as improving endothelial function as well as decreasing oxidative stress and inflammation [6]. In the context of cardiovascular disease, nitric oxide-derived oxidant species that promote atherogenesis are suppressed by statins [7]. Therefore, statins are considered as promising candidate agents for the prevention of CIN.

A number of studies have shown that statins may have a protective effect on CIN, but there remains uncertainty regarding time and way of administration of statins nor is it clear whether a specific statin is better than the others. Pairwise meta-analyses combine the results of homogeneous studies conducted on the same topic, whereas network meta-analyses evaluate simultaneously both direct and indirect comparisons across trials sharing one or more common comparator [8, 9, 10]. The purpose of our work was thus to perform a systematic review including both pairwise and network meta-analysis in order to evaluate more accurately the effect of statins compared to placebo or standard therapies for the prevention of CIN.

**METHODS**

**Design**

The present review was conducted in keeping with the current guidelines from the Preferred Reporting Items for Systematic Reviews and Meta-Analyses (PRISMA) group and from the Cochrane Collaboration. All reviewing activities were conducted by two independent reviewers (MP, GBZ), with divergences solved after consensus.

**Search**

MEDLINE/PubMed was searched for suitable studies according to Biondi-Zoccai et al, with the following substring:[11] (statin* OR atorvastatin OR rosuvastatin OR simvastatin OR lovastatin OR fluvastatin) AND (contrast AND (nephropathy OR ((renal OR kidney) AND (injury OR damage)))). In addition, the Cochrane Library, Google Scholar and Scopus were searched for additional studies. No language restriction was enforced in order to minimized the risk of publication bias, actually leading to the inclusion of a study published only in Chinese [12]. Searches were last updated on November 14, 2013, with the exception of the MEDLINE/PubMed query, which was last performed on July 15, 2014.

**Selection**

Citations were initially screened at the title/abstract level. If potentially pertinent, they were then retrieved as full texts. Studies were included if randomized, allocating one or more groups to statin therapy, and reporting dichotomous outcomes relevant to the assessment of CIN. Accordingly, studies were excluded if not randomized, duplicates, focusing on non-statin strategies, or lacking suitable outcome details.

**Abstraction and validity**

Study, patient, and procedural features were systematically extracted, with a particular focus on treatment strategy, type and volume of contrast, and CIN-related outcomes. The primary end-point was the occurrence, at the longest in-hospital follow-up, of CIN, defined as a relative increase in serum creatinine ≥25% or an absolute increase in serum creatinine ≥0.5 mg/dL (44 µmol/L), typically 48-72 hours after the index procedure. In addition, the following endpoints were also collected at the longest in-hospital follow-up: absolute increase in serum creatinine ≥0.5 mg/dL (44 µmol/L); relative increase in serum creatinine ≥25%. Conversely, the longest available clinical follow-up (thus well beyond hospitalization, when available), was exploited to abstract data on all-cause death and need for dialysis or renal replacement therapy. Study validity was appraised in keeping with ongoing recommendations distinguishing several sources of bias typical of randomized trials [10].

**Analysis**

Categorical variables are described as n (%) and continuous variables as median. Pairwise meta-analysis was performed within a frequentist framework, computing odds ratios (OR, 95% confidence intervals) by means of a Mantel-Haenszel random-effect method [8]. Network meta-analysis was performed within a Bayesian framework computing odds ratios (95% credible intervals) and probability of being best of any given treatment (Pbest) with a fixed-effect hierarchical model, sampling posterior probabilities by means of Markov chain Monte Carlo (MCMC) methods with Gibbs sampling from 150,000 iterations obtained after a 50,000-iteration training phase [9, 10]. Convergence was appraised graphically according to Gelman and Rubin, and non-informative priors were used throughout. A fixed-effect model was used for network meta-analysis based on the star-shaped evidence network, which is typically analyzed with such an approach, and after having confirmed that model fit was not different between a random-effect and a fixed-effect model computing values for deviance information criterion (DIC). Pairwise heterogeneity was appraised with chi-squared tests, whereas network inconsistency was evaluated comparing the results stemming from consistent and inconsistent models [8, 10]. Small study effects (e.g. publication bias) were explored by visual inspection of funnel plots. Computations were performed with RevMan and WinBUGS [10].

**RESULTS**

From an initial set of 33,030 citations retrieved from multiple databases, a total of 30 articles were analyzed as full reports according to our explicit selection criteria, finally yielding 14 studies eligible for inclusion in our review (Figure 1). These trials included a total of 6,160 patients, according to the following evidence network (Figure 2; Tables 1-3): 4 studies (706 subjects) compared atorvastatin high dose versus atorvastatin low dose [12, 13, 14, 15], 5 (1246) compared atorvastatin high dose versus placebo or standard therapy without statins [16, 17, 18, 19, 20], 1 (192) compared atorvastatin high dose versus rosuvastatin high dose [21], 2 (3541) compared rosuvastatin high dose versus placebo or standard therapy without statins [4, 22] 1 (247) compared simvastatin high dose versus placebo or standard therapy without statins [23], and 1 (228) compared simvastatin high dose versus simvastatin low dose [24].

Studies were clinically diverse, being conducted in Asia and Europe, and included patients with diabetes mellitus (median prevalence 23%) or mild to moderate chronic kidney disease (median baseline serum creatinine 1.0 mg/dL 1 (88 µmol/L). Several types of iodinated contrast media were used, including iobitridol, iodixanol, iohexol, iopamidol, and iopromide (median volume 161 mL). Study quality was adequate in most cases, but satisfactory details on randomization procedures and thorough double-blinding were present only for 5 trials [16, 17, 18, 20, 23].

Pairwise meta-analysis for CIN were first conducted to explore for overall clinical effects (Online figures 1-13). These computations showed that statins at high dose were associated with a reduced risk of CIN (OR=0.47 [95% confidence interval 0.37-0.60], p for effect<0.001, p for heterogeneity=0.59, I-squared=0%), of an absolute increase in serum creatinine ≥0.5 mg/dL (OR=0.57 [0.32-1.02], p for effect=0.06, p for heterogeneity=0.26, I-squared=25%), and of a relative increase in serum creatinine ≥25% (OR=0.35 [0.17-0.69], p for effect=0.003, p for heterogeneity=0.57, I-squared=0%). In addition, statins at high dose were associated with trends toward reduced risks of in-hospital dialysis (OR=0.27 [0.07-1.09], p for effect=0.07, p for heterogeneity=1.0, I-squared=0%) and death at a median follow-up of 3 months (OR=0.80 [0.31-2.10], p for effect=0.65, p for heterogeneity=0.61, I-squared=0%).

Subsequently, network meta-analysis was performed for the risk of CIN, distinguishing atorvastatin high dose, atorvastatin low dose, rosuvastatin high dose, simvastatin high dose, and simvastatin low dose. These computations showed that atorvastatin high dose was the regimen which was most likely to be beneficial (OR versus placebo=0.49 [95% credible interval 0.32-0.74], Pbest=34%), followed by rosuvastatin high dose (OR versus placebo=0.49 [0.34-0.69], Pbest=34%). Head-to-head comparison of atorvastatin high dose versus rosuvastatin high dose suggested a very similar effect (OR=1.00 [0.61-1.64]). Data for simvastatin high dose were apparently favorable, but inconclusive (OR versus placebo=0.66 [0.14-3.04], Pbest=32%), whereas data for atorvastatin low dose and simvastatin low dose clearly not in favor of clinical use to prevent CIN (respectively OR versus placebo=1.49 [0.69-3.45], Pbest<0.1%, and OR versus placebo=2.25 [0.37-14.61], Pbest=0.2%). Additional network analyses for the other CIN endpoints, dialysis or death were not possible for the paucity of reported events.

Consistency of pairwise and network analyses was satisfactory, in light of the low I-squared estimates, and the similar results yielded by consistent, inconsistent, fixed-effect, and random-effect Bayesian models. Even sensitivity analysis excluding the largest study (Han et al) [4] confirmed in direction and magnitude of effects the overall analysis (Table 5). Finally, no clear evidence of small study effects was found at funnel plot inspection (Online figures 3, 6, 9, 11, and 13).

**DISCUSSION**

This review, the first in the literature exploiting the totality of the evidence base with the novel mixed treatment comparison approach for comparative effectiveness analysis, confirms that statins at high dose reduce the risk of CIN in patients undergoing coronary angiography or revascularization with current iodinated contrast media, with potentially beneficial effects also on the risk of in-hospital dialysis. Moreover, network meta-analysis suggests that atorvastatin and rosuvastatin at high dose represent the first choice regimens in order to achieve a consistent and beneficial preventive effect on CIN, and may actually halve its incidence.

Invasive angiography exploiting iodinated contrast media may be complicated in several cases by CIN, with a negative impact on prognosis, hospital stay and costs [25]. Accordingly, means to prevent it may yield important and clinically-relevant benefits. Yet, its pathophysiology remains unclear. It may be due to direct toxicity on the renal tubular epithelium, oxidative stress, ischemic injury, and renal tubular obstruction, as any change in nitric oxide, prostaglandins, endothelin and adenosine in the vaso-regulation pathway could contribute to worsen medullary ischemia [26]. Many preventive strategies and treatments have been proposed for CIN, although only few with a demonstrated clinical efficacy [25, 27, 28].

In addition to their impact on cholesterol, statins are known to have multiple non-lipid lowering (i.e. pleiotropic) effects, which include several mechanisms involving inflammation responses, endothelial function, oxidative stress and apoptotic pathways [17]. Preventing contrast-induced renal cell apoptosis seems to play an important role in statins effects on CIN. Because inflammation is unquestionably involved in the pathogenesis of kidney injury, its modulation could be part of the mechanism expounding the reduced incidence of CIN after treatment with statins. The antioxidant effect of statins in addition to other antioxidant compounds (sodium bicarbonate solution and N-acetylcysteine) seems to reduce the occurrence of CIN just through scavenging reactive oxygen species (ROS) [14]. Khanal et al firstly reported, in a retrospective study that evaluated a large cohort of patients who underwent PCI, that patients on chronic statin therapy before the procedure had a significantly lower incidence of CIN [29]. Thereafter, several prospective non randomized or randomized placebo controlled trials suggested that a short-term regimen of high-dose statins before and after contrast exposure, in addition to standard measures for preventing kidney deterioration, might decrease the incidence of CIN .Nevertheless, the type and the dose threshold of statins to reduce the risk of CIN remained uncertain.

Our meta analysis represents the first work to exploit network meta-analytic methods to compare different statins in their CIN preventive effects. It shows that atorvastatin and rosuvastatin, when administered at high dose, represents the most effective preventive strategy. Accordingly, the present work provides important findings in support of the favorable risk-benefit balance of statins to prevent CIN, as offering patients a short-term yet high-dose statin regimen before administration of iodinated contrast appears as a remarkably safe, simple and effective intervention.

Focusing on current pharmacopeia, all statins are administered in active form except as lovastatin and simvastatin which are given as a prodrug and converted in vivo in the liver. More of 90% of statins are bound to plasma proteins, but rosuvastatin has a lower rate of link (88%). This is due to the hydrophilicity features of this molecule limiting the need for its transport in the blood through albumin. Hydrophilic statins encompass acute pleiotropic effects. Moreover they do not undergo cytocrome P-450 3A4 metabolism in the liver. These features may explain, at least in part, potential differences between statins in CIN prevention. In addition, as CIN is a paradigm of mutual hypoxic and toxic renal parenchymal injury mediated, to large extent, by an increased production of mediators of inflammation and ROS, and clinical as well as experimental findings clearly illustrate that hypoxia and enhanced ROS formation within the kidney following contrast media administration play a critical role in the development of CIN [30]**,** we speculate that atorvastatin and rosuvastatin may be more effective than other statins probably due to their higher anti-inflammation and antioxidant features.

This work has several limitations, including those typical of systematic reviews, pairwise meta-analyses and mixed treatment comparisons [8, 10]. More poignantly to the current findings, we exploited an evidence network with a prevalent star shape. In addition, we relied on surrogate outcomes for renal function, which have been called into question for being overly sensitive and subject to substantial between- and within-individual variability. In particular, relative increases in serum creatinine ≥25% may occur in many patients irrespectively of the amount of administered contrast. In addition, no study focused on non-coronary contrast media administration, and thus the implications of our results for other imaging procedures (e.g. computed tomography) are open to individual interpretation. Finally, due to the lack of patient-level data, key moderators on the risk of CIN, such as means of contrast administration (intravenous versus intra-arterial), volume of contrast, type of contrast [25], and baseline use of statins, could not be appraised quantitatively.

**CONCLUSION**

Atorvastatin and rosuvastatin, when administered at high doses and before iodinated contrast administration, have a consistent and beneficial preventive effect on CIN and may actually halve its incidence.

**ACKNOWLEDGEMENTS**

Dr. Biondi-Zoccai was, at the time of the preparation of this manuscript, the Congdon Visiting Professor at the VCU Pauley Heart Center, Virginia Commonwealth University, Richmond, VA, USA.

**FUNDING/CONFLICTS OF INTEREST**

Drs. Biondi-Zoccai and De Luca have consulted and lectured for Astra Zeneca.

**REFERENCES**

1. J. Golshahi, H. Nasri, M. Gharipour, “Contrast-induced nephropathy; A literature review,” J Nephropathol, vol. 3, no. 2, pp. 51-56, 2014.

2. T. Senoo, M. Motohiro, H. Kamihata, et al, "Contrast-induced nephropathy in patients undergoing emergency percutaneous coronary intervention for acute coronary syndrome," Am J Cardiol, vol. 105, no. 5, pp. 624-628, 2010.

3. J. Wi, Y. G. Ko, J. S. Kim, et al, "Impact of contrast-induced acute kidney injury with transient or persistent renal dysfunction on long-term outcomes of patients with acute myocardial infarction undergoing percutaneous coronary intervention, " Heart, vol. 97, no. 21, pp. 1753-1757, 2011.

4. Y. Han, G. Zhu, L. Han, et al, "Short-Term Rosuvastatin Therapy for Prevention of Contrast-Induced Acute Kidney Injury in Patients with Diabetes and Chronic Kidney Disease," J Am Coll Cardiol, vol. 63, no. 1, pp. 62-70, 2013.

5. S. N. Heyman, J. Reichman, M. Brezis, "Pathophysiology of radiocontrast nephropathy: a role for medullary hypoxia," Invest Radiol, vol. 34, no. 11, pp. 685-691, 1999.

6. J. A. Farmer, "Pleiotropic effects of statins," Curr Atheroscler Rep, vol. 2, no. 3, pp. 208-217, 2000.

7. M. H. Shishehbor, R. J. Aviles, M. L. Brennan, et al, "Association of nitrotyrosine levels with cardiovascular disease and modulation by statin therapy," JAMA, vol. 289, no. 13, pp. 1675-1680, 2003.

8. T. Greco, G. Landoni, G. Biondi-Zoccai, et al, "A Bayesian network meta-analysis for binary outcome: how to do it," Stat Methods Med Res 2013 Oct 28 [Epub ahead of print].

9. F. D'Ascenzo, G. Biondi-Zoccai, "Network Meta-analyses: The "White Whale" for Cardiovascular Specialists," J Cardiothorac Vasc Anesth, vol. 28, no. 1, pp. 169-173, 2013.

10. G. Biondi-Zoccai, “Network Meta-Analysis: Evidence Synthesis with Mixed Treatment Comparison,” Hauppauge, NY: Nova Science Publishers, 2014.

11. G. Biondi-Zoccai, P. Agostoni, A. Abbate, et al, "A simple hint to improve Robinson and Dickersin's highly sensitive PubMed search strategy for controlled clinical trials," Int J Epidemiol, vol. 34, no. 1, pp. 224-225, 2005.

12. S. Cao, P. Wang, K. Cui, et al, "Atorvastatin prevents contrast agent-induced renal injury in patients undergoing coronary angiography by inhibiting oxidative stress," Nan Fang Yi Ke Da Xue Xue Bao, vol. 32, no. 11, pp. 1600-1602, 2012.

13. X. Zhou, Y. Z. Jin, Q. Wang, et al, "Efficacy of high dose atorvastatin on preventing contrast induced nephropathy in patients underwent coronary angiography," Zhonghua Xin Xue Guan Bing Za Zhi, vol. 37, no. 5, pp. 394-396, 2009.

14. H. Li, X. Li, H. Ma, et al, “Atorvastatin combining with probucol: a new way to reduce serum uric acid level during perioperative period of interventional procedure,” ScientificWorldJournal, vol. 2014, pp. 565367, 2014.

15. S. H. Jo, J. Y. Hahn, S. Y. Lee, et al, “High-dose atorvastatin for preventing contrast-induced nephropathy in primary percutaneous coronary intervention,” J Cardiovasc Med (Hagerstown), 2014 Jul 16. [Epub ahead of print].

16. A. Toso, M. Maioli, M. Leoncini, et al, "Usefulness of atorvastatin (80 mg) in prevention of contrast-induced nephropathy in patients with chronic renal disease," Am J Cardiol, vol. 105, no. 3, pp. 288-292, 2010.

17. C. Quintavalle, D. Fiore, F. De Micco, et al, " Impact of a high loading dose of atorvastatin on contrast-induced acute kidney injury," Circulation, vol. 126, no. 25, pp. 3008-3016, 2012.

18. G. Patti, E. Ricottini, A. Nusca, et al, "Short-term, high-dose Atorvastatin pretreatment to prevent contrast-induced nephropathy in patients with acute coronary syndromes undergoing percutaneous coronary intervention (from the ARMYDA-CIN [atorvastatin for reduction of myocardial damage during angioplasty--contrast-induced nephropathy] trial," Am J Cardiol, vol. 108, no. 1, pp. 1-7, 2011.

19. H. Ozhan, I. Erden, S. Ordu, et al, "Efficacy of short-term high-dose atorvastatin for prevention of contrast-induced nephropathy in patients undergoing coronary angiography," Angiology, vol. 61, no. 7, pp. 711-714, 2010.

20. W. Li, X. Fu, Y. Wang, et al, "Beneficial effects of high-dose atorvastatin pretreatment on renal function in patients with acute ST-segment elevation myocardial infarction undergoing emergency percutaneous coronary intervention," Cardiology, vol. 122, no. 3, pp. 195-202, 2012.

21. A. Kaya, M. Kurt I. H. Tanboğa, et al, “Rosuvastatin versus atorvastatin to prevent contrast induced nephropathy in patients undergoing primary percutaneous coronary intervention (ROSA-cIN trial),” Acta Cardiol, vol. 68, no. 5, pp. 489-94, 2013.

22. M. Leoncini, A. Toso, M. Maioli, et al, "Early high-dose rosuvastatin for contrast-induced nephropathy prevention in acute coronary syndrome. Results from Protective effect of Rosuvastatin and Antiplatelet Therapy On contrast-induced acute kidney injury and myocardial damage in patients with Acute Coronary Syndrome (PRATO-ACS Study)," J Am Coll Cardiol, vol. 63, no. 1, pp. 71-79, 2014.

23. S. H. Jo, B. K. Koo, J. S. Park, et al, "Prevention of radiocontrast medium-induced nephropathy using short-term high-dose simvastatin in patients with renal insufficiency undergoing coronary angiography (PROMISS) trial--a randomized controlled study," Am Heart J, vol. 155, no. 3, pp. 499.e1-e8, 2008.

24. J. Xinwei, F. Xianghua, Z. Jing, et al, "Comparison of usefulness of simvastatin 20 mg versus 80 mg in preventing contrast-induced nephropathy in patients with acute coronary syndrome undergoing percutaneous coronary intervention," Am J Cardiol, vol. 104, no. 4, pp. 519-524, 2009.

25. G. Biondi-Zoccai G, M. Lotrionte M, H. S. Thomsen HS, et al, “Nephropathy after administration of iso-osmolar and low-osmolar contrast media: evidence from a network meta-analysis,” Int J Cardiol, vol. 172, no. 2, pp. 375-80, 2014.

26. C. Haller, I. Hizoh I, "The cytotoxicity of iodinated radiocontrast agents on renal cells in vitro," Invest Radiol, vol. 39, no. 3, pp. 149-154, 2004.

27. A. Pisani, M. Sabbatini, E. Riccio, et al, "Effect of a recombinant manganese superoxide dismutase on prevention of contrast-induced acute kidney injury," Clin Exp Nephrol 2013 Jun 27 [Epub ahead of print].

28. J. G. Webb, G. E. Pate, K. H. Humphries, et al, "A randomized controlled trial of intravenous N-acetylcysteine for the prevention of contrast-induced nephropathy after cardiac catheterization: lack of effect," Am Heart J, vol. 148, no. 3, pp. 422-429, 2004.

29. S. Khanal, N. Attallah, D. E. Smith, et al, "Statin therapy reduces contrast-induced nephropathy: an analysis of contemporary percutaneous interventions," Am J Med, vol. 118, no. 8, pp. 843-849, 2005.

30. R. E. Katholi, W. T.Woods Jr., G. J. Taylor, et al, "Oxygen free radicals and contrast nephropathy,” Am J Kidney Dis, vol. 32, no. 1, pp. 64-71, 1998.

**Table 1.** Included studies.

| **First author** | **Year** | **Journal** | **Patients** | **Multicenter setting** | **Location** | **Follow-up (days)** | **Selection criteria** |
| --- | --- | --- | --- | --- | --- | --- | --- |
| Cao | 2012 | Nan Fang Yi Ke Da Xue Xue Bao | 180 | No | China | 3 | ACS undergoing coronary angiography or PCI without renal failure |
| Han | 2013 | J Am Coll Cardiol | 2998 | Yes | China | 30 | Type 2 DM and stage 2-3 CKD |
| Jo | 2008 | Am Heart J | 247 | Yes | Korea | 180 | CKD, statin naive, undergoing coronary angiography |
| Jo | 2014 | J Cardiovasc Med | 218 | Yes | Korea | 180 | STEMI undergoing emergency PCI irrespective of renal dysfunction |
| Kaya | 2013 | Acta Cardiol | 192 | No | Turkey | 2 | STEMI undergoing emergency PCI without renal dysfunction |
| Leoncini | 2013 | J Am Coll Cardiol | 543 | No | Italy | 180 | NSTE-ACS, statin naive, selected for early invasive strategy, without acute or end-stage renal failure |
| Li | 2012 | Cardiology | 161 | No | China | 30 | STEMI undergoing emergency PCI without renal dysfunction |
| Li | 2014 | ScientificWorldJournal | 208 | No | China | 1 | Patients undergoing coronary angiography or angioplasty |
| Ozhan | 2010 | Angiology | 130 | No | Turkey | 2 | Patients undergoing coronary angiography without renal insufficiency (serum creatinine <1.5 mg/dl) |
| Patti | 2011 | Am J Cardiol | 241 | Yes | Italy | 2 | NSTE-ACS, statin naive, receiving early PCI |
| Quintavalle | 2012 | Circulation | 410 | Yes | Italy | 365 | CKD, statin naive, scheduled for elective coronary angiography or PCI |
| Toso | 2010 | Am J Cardiol | 304 | No | Italy | 30 | CKD, statin naive, without end-stage renal failure requiring dialysis |
| Xinwei | 2009 | Am J Cardiol | 228 | No | China | 7 | ACS undergoing PCI |
| Zhou | 2009 | Zhonghua Xin Xue Guan Bing Za Zhi | 100 | No | China | 3 | Coronary angiography or PCI |

ACS=acute coronary syndrome; CKD=chronic kidney disease; DM=diabetes mellitus; NSTE-ACS=non-ST-elevation ACS; PCI=percutaneous coronary intervention; STEMI=ST-elevation myocardial infarction

**Table 2.** Patient and procedural features.

| **First author** | **Year** | **Experimental therapy** | **Control therapy** | **Contrast type** | **Median contrast volume** | **Age (years)** | **Diabetes mellitus** | **Baseline serum creatinine (mg/dL)** |
| --- | --- | --- | --- | --- | --- | --- | --- | --- |
| Cao | 2012 | Atorvastatin 40 mg/day started 3 days before angioplasty followed by atorvastatin 20 mg/day | Atorvastatin 20 mg/day | NA | 161 | 63 | 20% | NA |
| Han | 2013 | Rosuvastatin 10 mg/day from 2 days before to 3 days after contrast | No statin | Iodixanol | 115 | 61 | 100% | 1.1 |
| Jo | 2008 | Simvastatin 40+40 mg before angiography followed by simvastatin 40+40 mg afterwards | Placebo | Iodixanol | 182 | 65 | 26% | 1.2 |
| Jo | 2014 | Atorvastatin 80 mg before angiography followed by atorvastatin 80 mg/day for 5 days and then 10 mg/day | Atorvastatin 10 mg/day | NA | NA | 60 | 26% | 1.1 |
| Kaya | 2013 | Atorvastatin 80 mg before angiography | Rosuvastatin 40 mg before angiography | Iopromide | 153 | 63 | 20% | 0.9 |
| Leoncini | 2013 | Rosuvastatin 40 mg at-admission followed by 20 mg/day | No statin | Iodixanol | 261 | 66 | 21% | 1.0 |
| Li | 2012 | Atorvastatin 80 mg at admission followed by atorvastatin 40 mg after angiography | Placebo | Iopromide | 102 | 66 | 28% | 0.9 |
| Li | 2014 | Atorvastatin 40 mg before angiography, followed by atorvastatin 40 mg/day | Atorvastatin 20 mg/day | Iopamidol | 186 | 61 | 24% | 0.9 |
| Ozhan | 2010 | Atorvastatin 80 mg before angiography, followed by atorvastatin 80 mg 48 hours after contrast administration | No statin | Iopamidol | 95 | 55 | 16% | 0.8 |
| Patti | 2011 | Atorvastatin 80 mg 12 hrs before angiography; further 40 mg pre-procedure (2 hrs before), followed by atorvastatin 40 mg/day | Placebo | Iobitridol | 211 | 66 | 27% | 1.0 |
| Quintavalle | 2012 | Atorvastatin 80 mg (within 24 hrs before contrast exposure), followed by atorvastatin 20 mg/day | No statin | Iodixanol | 180 | 70 | 41% | 1.3 |
| Toso | 2010 | Atorvastatin 80 mg 48 hours before and 48 hours after contrast administration | Placebo | Iodixanol | 157 | 75 | 21% | 1.2 |
| Xinwei | 2009 | Simvastatin 80 mg from admission to day before PCI, followed by simvastatin 20 mg/day | Simvastatin 20 mg/day | Iodixanol or iohexol | 233 | 65 | 21% | 0.8 |
| Zhou | 2009 | Atorvastatin 80 mg/day before the procedure, 10 mg/day for 6 days after procedure | Atorvastatin 10 mg/day for 7 days | Iopamidol | 116 | 61 | 20% | 1.1 |

NA=not available or applicable

**Table 3.** Internal validity of included studies.

| **First author** | **Year** | **Inadequate allocation sequence generation?** | **Inadequate allocation concealment?** | **Inadequate blinding?** | **Incomplete outcome data?** | **Selective outcome reporting?** | **Risk of other bias?** |
| --- | --- | --- | --- | --- | --- | --- | --- |
| Cao | 2012 | Unclear | Unclear | Unclear | No | Yes (lack of some individual CIN data) | No |
| Han | 2013 | No | No | Yes (open-label) | No | Yes (lack of some individual CIN data) | No |
| Jo | 2008 | No | No | No | No | Yes (lack of some individual CIN data) | No |
| Jo | 2014 | No | Unclear | Yes (open-label) | No | Yes (lack of some individual CIN data) | No |
| Kaya | 2013 | Unclear | Unclear | Yes (open-label) | No | Yes (lack of some individual CIN data) | No |
| Leoncini | 2013 | No | Yes (open-label list) | Yes (open-label) | No | Yes (lack of some individual CIN data) | No |
| Li | 2012 | No | No | No | No | Yes (lack of some individual CIN data) | No |
| Li | 2014 | Unclear | Unclear | Yes (open-label) | No | Yes (lack of some individual CIN data) | No |
| Ozhan | 2010 | Unclear | Unclear | Unclear | No | Yes (lack of some individual CIN data) | No |
| Patti | 2011 | No | No | No | No | Yes (lack of some individual CIN data) | No |
| Quintavalle | 2012 | No | No | No | No | Yes (lack of some individual CIN data) | No |
| Toso | 2010 | No | No | No | No | Yes (lack of some individual CIN data) | No |
| Xinwei | 2009 | No | Yes (open-label study) | Yes (open-label) | No | Yes (lack of some individual CIN data) | No |
| Zhou | 2009 | Unclear | Unclear | Unclear | No | Yes (lack of some individual CIN data) | No |

CIN=contrast-induced nephropathy

**Table 4.** Effect estimates for the risk of contrast-induced nephropathy. Results are reported as probability of being best treatment (Pbest) and odds ratios (OR) with 95% credible intervals of a given row item versus a corresponding column item. Thus, OR <1 indicates lower risk in the corresponding row item and OR >1 indicates lower risk in the corresponding column item.

| **Treatment** | **Atorvastatin high dose** | **Atorvastatin low dose** | **Rosuvastatin high dose** | **Simvastatin high dose** | **Simvastatin low dose** | **Placebo or no statin** |
| --- | --- | --- | --- | --- | --- | --- |
| **Atorvastatin high dose** | Pbest=34% | ***OR=0.33 (0.16-0.63)*** | OR=1.00 (0.61-1.64) | OR=0.69 (0.12-4.35) | OR=0.22 (0.03-1.35) | ***OR=0.49 (0.32-0.74)*** |
| **Atorvastatin low dose** | ***OR=3.05 (1.59-6.21)*** | Pbest<0.1% | ***OR=3.85 (1.27-14.29)*** | OR=2.86 (0.41-24.39) | OR=0.67 (0.09-5.01) | OR=1.49 (0.69-3.45) |
| **Rosuvastatin high dose** | OR=1.00 (0.61-1.63) | ***OR=0.26 (0.07-0.79)*** | Pbest=34% | OR=0.76 (0.14-4.55) | OR=0.73 (0.16-3.70) | ***OR=0.49 (0.34-0.69)*** |
| **Simvastatin high dose** | OR=1.44 (0.23-8.11) | OR=0.35 (0.04-2.42) | OR=1.32 (0.22-7.13) | Pbest=32% | ***OR=0.29 (0.10-0.72)*** | OR=0.66 (0.14-3.04) |
| **Simvastatin low dose** | OR=4.61 (0.74-30.37) | OR=1.50 (0.20-10.86) | OR=1.37 (0.27-6.42) | ***OR=3.40 (1.38-9.88)*** | Pbest=0.2% | OR=2.25 (0.37-14.61) |
| **Placebo or**  **no statin** | ***OR=2.05 (1.35-3.09)*** | OR=0.67 (0.29-1.44) | ***OR=2.04 (1.44-2.94)*** | OR=1.51 (0.33-7.14) | OR=0.44 (0.07-2.70) | Pbest=0 |

**Table 5.** Effect estimates for the risk of contrast-induced nephropathy excluding the study by Han et al. Results are reported as probability of being best treatment (Pbest) and odds ratios (OR) with 95% credible intervals of a given row item versus a corresponding column item. Thus, OR <1 indicates lower risk in the corresponding row item and OR >1 indicates lower risk in the corresponding column item.

| **Treatment** | **Atorvastatin high dose** | **Atorvastatin low dose** | **Rosuvastatin high dose** | **Simvastatin high dose** | **Simvastatin low dose** | **Placebo or no statin** |
| --- | --- | --- | --- | --- | --- | --- |
| **Atorvastatin high dose** | Pbest=20% | ***OR=0.32 (0.15-0.65)*** | OR=1.22 (0.66-2.33) | OR=0.65 (0.15-3.85) | OR=0.19 (0.03-1.37) | ***OR=0.47 (0.31-0.70)*** |
| **Atorvastatin low dose** | ***OR=3.12 (1.53-6.73)*** | Pbest<0.1% | ***OR=3.85 (1.47-11.09)*** | OR=2.08 (0.36-14.08) | OR=0.60 (0.08-4.98) | OR=1.45 (0.62-3.45) |
| **Rosuvastatin high dose** | OR=0.82 (0.43-1.51) | ***OR=0.26 (0.10-0.68)*** | Pbest=34% | OR=0.53 (0.12-3.23) | OR=0.16 (0.02-1.22) | ***OR=0.38 (0.22-0.65)*** |
| **Simvastatin high dose** | OR=1.54 (0.26-6.87) | OR=0.48 (0.07-2.77) | OR=1.90 (0.31-8.56) | Pbest=20% | ***OR=0.29 (0.10-0.78)*** | OR=0.72 (0.13-3.06) |
| **Simvastatin low dose** | OR=5.24 (0.73-32.12) | OR=1.67 (0.20-12.66) | OR=6.46 (0.82-41.28) | ***OR=3.42 (1.28-10.03)*** | Pbest=0.2% | OR=2.46 (0.35-14.17) |
| **Placebo or**  **no statin** | ***OR=2.15 (1.42-3.28)*** | OR=0.69 (0.29-1.61) | ***OR=2.63 (1.54-4.55)*** | OR=1.39 (0.33-7.69) | OR=0.41 (0.07-2.86) | Pbest=0 |

**FIGURE 1.** Review profile.

**FIGURE 2.** Evidence network. Continuous lines represent head-to-head randomized comparisons (the thickness of the line corresponding to the number of trials) with both direct and indirect effect estimates, whereas dashed lines represent only indirect effect estimates. The thickness of the rectangles corresponds to the patients receiving a specific treatment.

**FIGURE 3.** Forest plot summarizing risk estimates stemming from network meta-analysis comparing different statin regimens for the risk of contrast-induced nephropathy. OR=odds ratio. CI=confidence interval; SE=standard error.
