# Supplementary material for: A Network Meta-Analysis on Randomized Trials Focusing on the Preventive Effect of Statins on Contrast-Induced Nephropathy
Source: Biomed Res Int. 2014 Sep 7;2014:213239. doi: 10.1155/2014/213239 (PMC4170696; doi:10.1155/2014/213239)
Supplement: Supplementary file 1 — The Supplementary Material includes complementary information for the pairwise and sensitivity analyses. [file 213239.f1.zip › supplementary material/1048284.doc]

**Online supplement for Peruzzi et al: A network meta-analysis on randomized trials focusing on the preventive effect of statins on contrast-induced nephropathy**

**Online figure 1.** Forest plot for the risk of contrast-induced nephropathy. CI=confidence interval; df=degrees of freedom; M-H=Mantel-Haenszel; I-squared=statistical inconsistency.

**Online figure 2.** Forest plot for the risk of contrast-induced nephropathy, excluding Kaya et al [21]. CI=confidence interval; df=degrees of freedom; M-H=Mantel-Haenszel; I-squared=statistical inconsistency.

**Online figure 3.** Funnel plot for the risk of contrast-induced nephropathy. OR=odds ratio; SE=standard error.

**Online figure 4.** Forest plot for the risk of a relative increase in serum creatinine ≥25%. CI=confidence interval; df=degrees of freedom; M-H=Mantel-Haenszel; I-squared=statistical inconsistency.

**Online figure 5.** Forest plot for the risk of a relative increase in serum creatinine ≥25% excluding Kaya et al [21]. CI=confidence interval; df=degrees of freedom; M-H=Mantel-Haenszel; I-squared=statistical inconsistency.

**Online figure 6.** Funnel plot for the risk of a relative increase in serum creatinine ≥25%. OR=odds ratio; SE=standard error.

**Online figure 7.** Forest plot for the risk of an absolute increase in serum creatinine ≥0.5 mg/dL. CI=confidence interval; df=degrees of freedom; M-H=Mantel-Haenszel; I-squared=statistical inconsistency.

**Online figure 8.** Forest plot for the risk of an absolute increase in serum creatinine ≥0.5 mg/dL excluding Kaya et al [21]. CI=confidence interval; df=degrees of freedom; M-H=Mantel-Haenszel; I-squared=statistical inconsistency.

**Online figure 9.** Funnel plot for the risk of an absolute increase in serum creatinine ≥0.5 mg/dL. OR=odds ratio; SE=standard error.

**Online figure 10.** Forest plot for the risk of in-hospital dialysis. CI=confidence interval; df=degrees of freedom; M-H=Mantel-Haenszel; I-squared=statistical inconsistency.

**Online figure 11.** Funnel plot for the risk of in-hospital dialysis. OR=odds ratio; SE=standard error.

**Online figure 12.** Forest plot for the risk of death after a median follow-up of 2 months. CI=confidence interval; df=degrees of freedom; M-H=Mantel-Haenszel; I-squared=statistical inconsistency.

**Online figure 13.** Funnel plot for the risk of death after a median follow-up of 2 months. OR=odds ratio; SE=standard error.
